# Better detoxifying effect of ripe forsythiae fructus over green forsythiae fructus and the potential mechanisms involving bile acids metabolism and gut microbiota

**DOI:** 10.3389/fphar.2022.987695

**Published:** 2022-08-12

**Authors:** Tao Wang, Xu-Jiong Li, Ling-Hao Qin, Xue Liang, Huan-Huan Xue, Jing Guo, Shi-Fei Li, Li-Wei Zhang

**Affiliations:** ^1^ Institute of Molecule Science, Modern Research Center for Traditional Chinese Medicine, Key Laboratory of Chemical Biology and Molecular Engineering of Ministry of Education, Shanxi University, Taiyuan, China; ^2^ Department of Pharmacy, Changzhi Medical College, Changzhi, China; ^3^ Department of Physiology, Changzhi Medical College, Changzhi, China; ^4^ School of Pharmacy, Guangdong Pharmaceutical University, Guangzhou, China

**Keywords:** forsythiae fructus, bile duct ligation, detoxifying, bile acids, gut microbiota

## Abstract

Forsythiae Fructus (FF), the fruit of *Forsythia suspensa* (Thunb.) Vahl. (Lianqiao), is one of the most fundamental herbs in Traditional Chinese Medicines (TCM), mainly due to its heat-clearing and detoxifying effects. There are two types of FF, the greenish fruits that start to ripen (GF) and the yellow fruits that are fully ripe (RF), called “Qingqiao” and “Laoqiao” referred to the Chinese Pharmacopoeia, respectively. It undergoes a complex series of changes during the maturation of FF. However, the clinical uses and preparation of phytopharmaceuticals of FF have not been distinguished to date. Moreover, there is limited information on the study of the difference in pharmacological activity between RF and GF. In this study, a rat model of bile duct ligation (BDL)-induced cholestasis was used to compare the differences in their effects. RF was found to have better results than GF in addressing toxic bile acids (BAs) accumulation and related pathological conditions caused by BDL. The underlying mechanism may be related to the interventions of gut microbiota. The results of the present study suggest that the better detoxifying effect of RF than GF may be indirectly exerted through the regulation of gut microbiota and thus the improvement of BAs metabolism.

## Introduction

*Forsythia suspensa* (Thumb.) Vahl. (Family Oleaceae) is widely distributed in China, Korea, Japan, and many European nations (Li et al., 2014). The dried fruit of the plant, Forsythiae Fructus (FF), named “Lianqiao” in Chinese, is one of the most recognized traditional Chinese medicines (TCM) due to its removal effects of heat and toxins (Bao et al., 2016). It has been used for anti-inflammatory, antioxidant, antiendotoxin, antimicrobial and antiviral purposes (Lu et al., 2010; Li and Peng, 2013; Kuo et al., 2014; Lee et al., 2016; Law et al., 2017). FF is listed as an official drug in Chinese, Korean, and Japanese Pharmacopoeias (Nishibe, 2002). In addition, FF has been studied and developed as a dietary supplement for food and feed considering its nutritional properties (Lu et al., 2010; Jeong et al., 2021).

According to the maturity level, FF could be classified into green Forsythiae Fructus (GF, called “Qingqiao” in Chinese) and ripe Forsythiae Fructus (RF, called “Laoqiao” in Chinese). Both of them are official sources of FF (Nishibe, 2002; Qu et al., 2008; Xia et al., 2009). F. suspensa undergoes a complex series of physical and biochemical changes during the ripening process from GF to RF. There must be differences between GF and RF in chemical constituents and biological activities. Studies showed that the contents of some representative constituents, such as forsythoside A, phillyrin, and rutin, were significantly higher in GF than in RF (Bai et al., 2015). In fact, GF is more frequently selected as a raw material for the production of herbal preparations containing FF in China. As a result, GF is often over-harvested, which in turn reduces the supply of RF. However, RF was preferred for traditional Chinese formula and export market (Qu et al., 2017). Thus, how to choose between RF and GF is still an open question, and the clinical applications of the two have not been distinguished to date. Consequently, evaluating the differences between RF and GF is both necessary and urgent (Wang et al., 2018).

In our previous investigations, eight constituents were found to be different between RF and GF. Furthermore, the antioxidant activity of GF was higher than that of RF (Jia et al., 2015). However, *in vivo* experiments on the comparison of the two have not been conducted. As a continuing program aimed at discovering the distinction between RF and GF, an animal model suitable for the comparative evaluation is urgently needed.

Obstruction of the biliary flow into the duodenum, accumulation of bile salts in the liver cells and biliary tract are known as cholestasis. As a result of cholestasis, some pathological changes occur in the organism. Some of the predominant ones at early stage include increased bile salt concentrations in plasma and hepatocyte, and decreased bile acids (BAs) in intestinal lumen (Sarac et al., 2015). The former causes pruritus, hepatocellular toxic injury and progressive hepatic fibrogenesis. While, the latter leads to bacterial proliferation, disruption of intestinal integrity, bacterial translocation and endotoxemia (Erenoğlu et al., 2011; Haque and Barritt Iv, 2016). These pathophysiological disturbances associated with cholestatic liver disease were discovered in both humans and experimental animals. A well-established experimental animal model of extrahepatic cholestasis is bile duct ligation (BDL) in rodents (Gujral et al., 2003; Pavlidis and Pavlidis, 2018). Inflammation, oxidative stress, elevated endotoxin levels and intestinal dysbacteriosis are important features of BDL model (Hong et al., 2007; Wiest et al., 2017; Tan et al., 2018). As a traditional herbal medicine, FF has effects of antifebrile, detoxification, detumescence, liver protection and cholagogue (Nishibe, 2002; Wang et al., 2018). Modern pharmacological researches also indicated that the extracts or the components of FF possessed anti-inflammatory, anti-oxidant, anti-bacterial, anti-endotoxin, and hepatobiliary protective activities (Lu et al., 2010; Kuo et al., 2014; Bao et al., 2016; Lee et al., 2016; Wang et al., 2016; Zhao et al., 2017; Qin et al., 2020). Given the many correspondences between the pathological features of the BDL model and the pharmacological effects of FF, we attempted to apply this model to the comparative evaluation of RF and GF.

**FIGURE 1:**
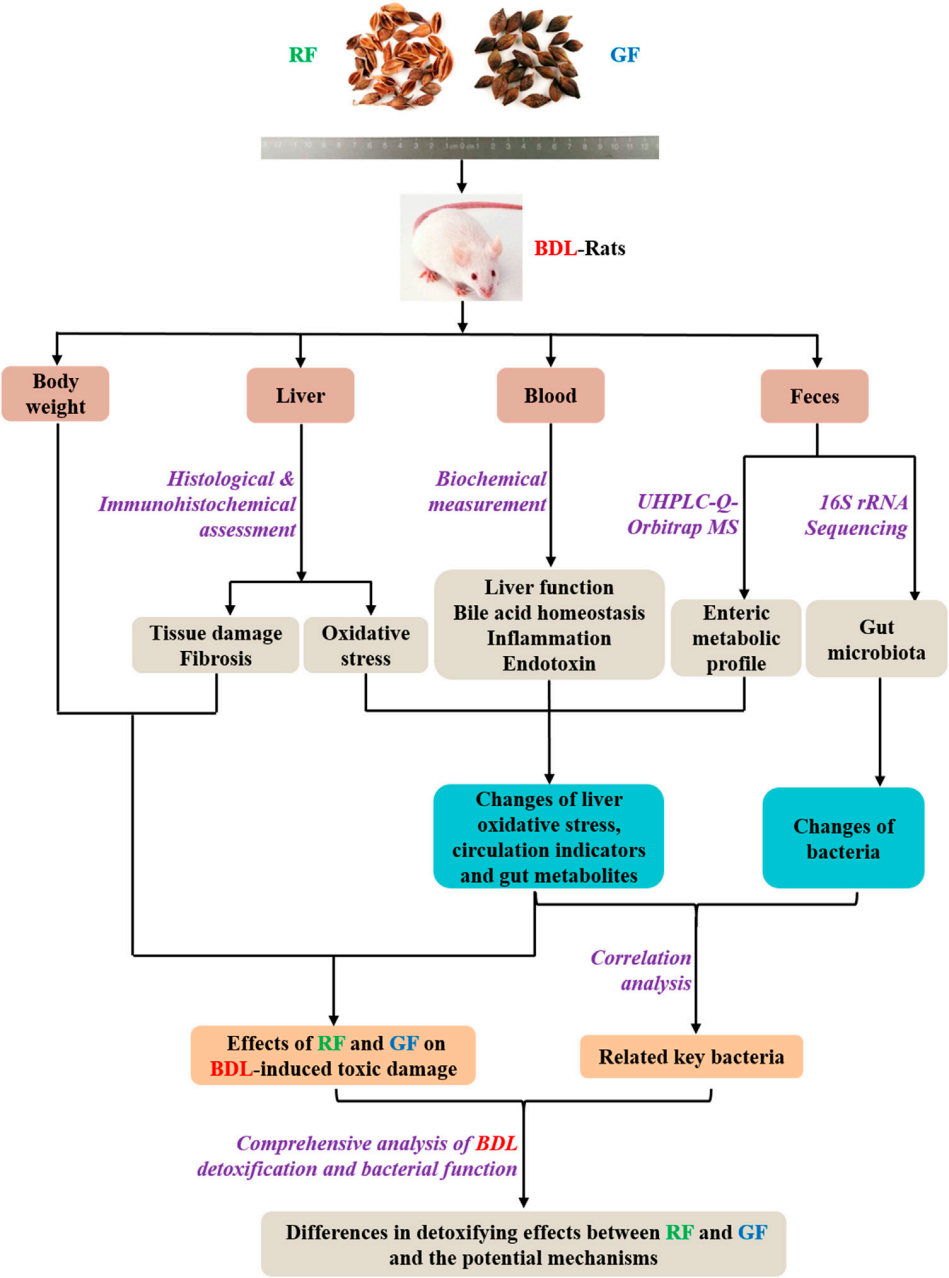
Research scheme for investigating the differences in detoxifying effects between RF and GF and the potential mechanisms.

In our preliminary experiments, RF and GF showed different trends in their effects on BDL animals. In view of this, more in-depth research needs to be carried out. The purpose of this study was to evaluate the similarities and differences of therapeutic effects between RF and GF, as well as the underlying mechanism. The study was designed as shown in Figure 1. We hope the results could provide references for the correct selection and reasonable application of FF.

## Materials and methods

### Preparation of herbal extracts

RF and GF samples were collected from Changzhi, Shanxi province of China, which is considered the main producing area of FF. The samples were authenticated by L-WZ of Shanxi University. All voucher specimens were deposited in the Institute of Molecular Science, Shanxi University, Taiyuan, China. All samples were ground to a fine powder and sieved with a bolt (20 meshes). RF and GF power (100 g) were respectively refluxed with 1,000 ml distilled water for 1 h, 2 times. The twice-extracted decoctions were combined and concentrated to generate suspensions of RF and GF (1.0 g/ml, which were relative to the quantity of the dried herbal powder).

### Animal treatment

Male Sprague-Dawley rats (180–200 g, 5–6 weeks-of-age, SPF) were purchased from Si Pei Fu biotech Ltd. (Beijing, China). Animals were provided chow and water *ad libitum*, and maintained in a 12 h light/dark cycle for 1 week prior to experimentation. All rats were cared carefully under a protocol that was in accordance with institutional guidelines for animal research and was approved by the Ethics Committee of Changzhi Medical College (NO. DW2021048). Efforts were made to minimize animal suffering and reduce the number of animals used in experimental groups.

A total of 24 rats were randomly divided into four groups with 6 animals in each: group of Sham operation (Sham Group), group of bile duct ligation (BDL Group), group of bile duct ligation treated with RF (BDL_RF Group) and group of bile duct ligation treated with GF (BDL_GF Group). Under ketamine anesthesia (100 mg/kg, intramuscular), the rats in BDL, BDL_RF, and BDL_GF groups underwent common bile duct ligation (BDL) as described in our previous research (Li et al., 2017). Briefly, the common bile duct was doubly ligated by 3/0 silk thread. Then, the abdominal layers were closed one by one after common bile duct was cut in the middle of the double ligation thread. Common bile duct was left without ligation in rats of the Sham group, and the remaining operations were the same as in the other groups. One day after BDL surgery, BDL_RF and BDL_GF groups were administrated (i.g.) 1.35 ml/kg (calculated according to the recommended dose of Chinese Pharmacopoeia) RF and GF extraction solution for 3 weeks respectively. Sham and BDL groups were i.g. administrated 1.35 ml/kg physiological saline. The body weight of each animal was assessed every day.

Three weeks after surgery, fecal and blood samples were collected from each rat. The feces were collected and transferred to sterilized airtight vials and weighted within 30 min, then immediately snap frozen with liquid nitrogen and stored in −80°C until use. Blood was centrifuged at 3,000 rpm for 10 min at 4°C to obtain the serum and stored at −20°C for further testing. Liver tissues were harvested, rinsed in cold isotonic saline and either fixed with 4% paraformaldehyde or immediately frozen in liquid nitrogen for further analysis.

### Histological assessment of liver

Liver samples of rats were fixed in 10% formalin solution. Following dehydration in a gradient ascending ethanol, the liver tissues were cleared in xylene and embedded in paraffin. Then, the tissues were cut into 5 μm sections and prepared for HE and Masson staining. The obtained histological slides were examined under a light microscope (Olympus CH20, Olympus, Japan).

### Immunohistochemical and oxidative stress assessment of liver

For immunohistochemical analysis, liver sections (4-μm thick) were prepared from paraffin-embedded specimens, using routine methods. Endogenous peroxidase was inactivated by treating sections with 3% H_2_O_2_ at room temperature for 15 min. After blocking with goat serum for 1 h, anti-Nrf2 antibody (1: 200, ab31163, Abcam, United States) was added for incubation at 4°C overnight. The slides were then incubated with a secondary antibody for 1 h at room temperature and visualized using DAB reagent. Yellow and brown staining in the nucleus and cytoplasm were considered positive. For measurement of oxidative stress indicators, accurately weighed liver tissue was rapidly homogenized with cold saline, centrifuged, and the supernatant was taken as the liver homogenate to be tested. The levels of SOD and MDA in livers were measured using MDA and SOD assay kits (Nanjing Jiancheng, China).

### Biochemical measurement of serum

Commercial ELISA kits (Shanghai MlBio, China) of ALP, ALT, AST,γ-GT, IL-1β, IL-6, TNF-α and ET were used to determine the serum levels of these biochemical indicators according to the manufacturer’s instructions. TBA, TBIL and DBIL levels were measured using commercial colorimetric assay kits (Abcam, United States) in accordance with the instructions.

### Enteric metabolomics analysis

Fecal samples were immersed in 0.1% formic acid acetonitrile 3 times their total weight, and then vortexed for 1 min and ultrasonic-extracted for 10 min. The extracts were centrifuged at 12,000 rpm for 10 min at 4°C. The supernatant was filtered by 0.22 μm ultrafiltration membrane for LC-MS analysis. In addition, to investigate the stability of instruments and analytical methods, QC sample was prepared through combining 50 μL of each analyzed sample, and analyzed per 4 samples during the whole analysis process.

The metabolomics profiling was accomplished using a UHPLC Q-Exactive Orbitrap mass spectrometer (Thermo Fisher, United States) equipped with a heated electrospray ionization (HESI) probe. Acquisition mode, positive and negative ion switching; scan mode, full scan/dd-MS2; m/z acquisition range, 100–1,500; positive electrode spray voltage, 3.5 kV; negative electrode spray voltage, 2.5 kV; capillary temperature, 320°C; probe heater temperature, 300°C; sheath gas flow rate, 35 arb; auxiliary gas flow rate, 10 arb; resolution setting, MS full scan 35,000 FWHM and MS/MS 17500 FWHM; NCE settings, 20, 40, 60 eV.

Chromatography separations were performed on a Waters ACQUITY UPLC HSS T3 column (2.1 × 100 mm, 1.7 μm, Waters, United States). Mobile phase A, 0.1% formic acid water; mobile phase B, acetonitrile; flow rate, 0.2 ml min-1; injection volume, 5 μL; column temperature, 45°C. The mobile phase gradient was as follows: 0–2 min, 2% B; 2–3 min, 2–40% B; 3–5 min, 40–43% B; 5–11 min, 43%–50% B; 11–18 min, 50–70% B; 18–23 min, 70–98% B; 23–24 min, 98% B; 24–24.5 min, 98–2% B; 24.5–27 min, 2% B.

The LC–MS raw data were exported using an Xcalibur workstation (Thermo Fisher, United States), and then imported to Compound Discoverer 3.1 (Thermo Fisher, United States) to obtain the aligned peak data. The parameters were set as follows: quality range, 100–1,500; mass tolerance, 5 ppm; retention time tolerance, 0.2 min; SNR threshold, 3. All integrated spectra were normalized to their total areas to eliminate the concentration differences. Then, metabolite peaks were introduced into Simca-P 13.0 software (Umetric, Sweden) for PCA and OPLS-DA. The quality of OPLS-DA model was validated by computing the cross-validation (double cross validation) parameter Q^2^. The value of Q^2^ close to 1 indicated an excellent predictability of the model. S-plots constructed using OPLS-DA were applied to select the potential variables for differentiation. VIP values presented the impact of each metabolite in the OPLS-DA model. Metabolite peaks were then assigned by molecular weights, MS/MS, and elemental compositions, and interpreted with available biochemical databases, such as HMDB (http://www.hmdb.ca/), KEGG (https://www.kegg.jp/), METLIN (http://metlin.scripps.edu/) and Chemspider (http://www.chemspider.com/).

### Microbiota analysis

Fecal genomic DNA was extracted and purified using Fast DNA SPIN extraction kits (MP Biomedicals, United States). Agarose gel electrophoresis was performed to assess the quality of the extracted DNA. The V3-V4 region of bacterial 16S rRNA gene was amplified. AMPure XP Beads (Beckman Coulter, United States) was used to purify amplicons, followed by quantification using Quant-iT PicoGreen dsDNA Assay Kit (Invitrogen, United States). Purified amplicons were mixed in equal amounts, and 2 × 300-bp pair-end sequencing was conducted on Illumina MiSeq platform. QIIME (version 1.80) was employed to process the raw data. High-quality sequences were clustered into operational taxonomic units (OTUs) at 97% sequence identity. The sequence with the highest abundance in each OTU was selected as the representative sequence for that OTU. Taxonomic classification was carried out by comparing OUT representative sequences with template sequences from Greengenes databases. The abundance matrix after removal of rare OTUs with abundances below 0.001% was used for subsequent comparative analysis between groups and visualization. Alpha diversity and taxa summaries were created through QIIME with sequencing depth and maximum rarefaction depth. The sequencing datasets are deposited in NCBI (https://www.ncbi.nlm.nih.gov/sra/PRJNA837391). The microbial community analysis was performed by Shanghai Personal Biotechnology Co., Ltd. (Shanghai, China).

### Statistical analysis

All the bar plots in this study were generated with Prism 8.0 software (GraphPad, United States). Statistical differences between groups were analyzed by one-way analysis of variance (ANOVA) followed by Tukey’s multiple comparisons test. Differences of microbial data between multiple samples were assessed by Mann–Whitney *U*-test. Results are presented as mean ± SEM. *p* < 0.05 was considered statistically significant. Correlations were performed by one-tailed Spearman’s analysis with 95% confidence interval and heatmaps were constructed using the GENESCLOUD online platform of Personal Biotechnology (https://www.genescloud.cn/).

## Results

### Compared with GF, RF has a stronger effect on preventing the disease progress of bile duct ligation-rats

**FIGURE 2:**
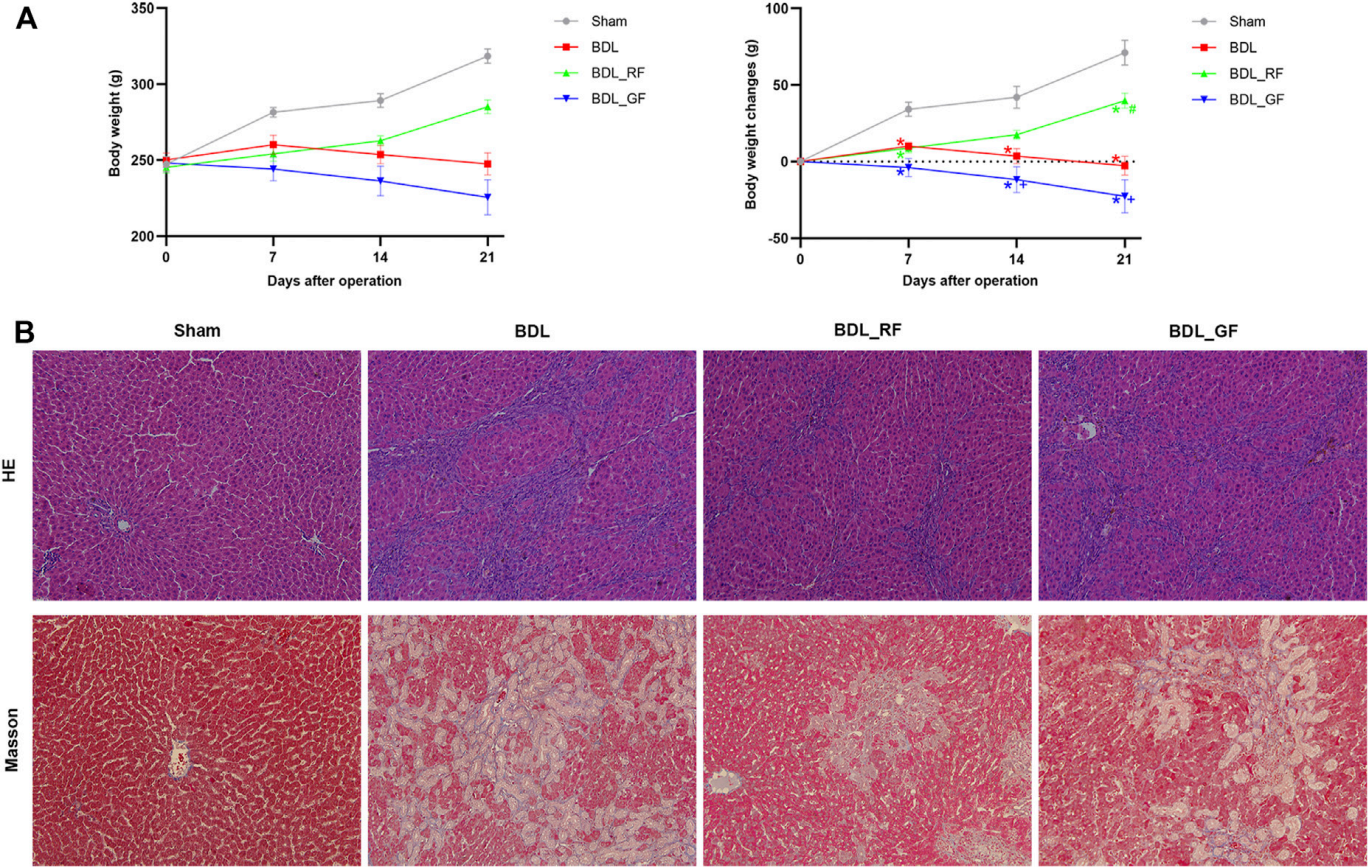
Effects of RF and GF on body weight and liver injury of BDL-rats. **(A)** Body weight changes; (*: *p* < 0.05, compared with the Sham group; #: *p* < 0.05, compared with the BDL group; +: *p* < 0.05, compared with the BDL_RF group, *n* = 6). **(B)** Representative histopathological findings of liver tissues stained with haematoxylin-eosin (HE) (×200 magnification) and Masson (×200 magnification).

After 21 days, the Sham group gained an average of 71 g of body weight, while the BDL group lost 3 g of body weight. The body weight changes of rats in BDL_RF and BDL_GF groups showed significant differences (*p* < 0.05, Figure 2A). The BDL_RF group gained 40 g of body weight, while the BDL_GF group lost 23 g of body weight. Haematoxylin-eosin (HE) staining results (Figure 2B) showed that the Sham group had intact hepatic lobules with a central vein surrounded by radially arranged hepatocyte cords. Disorganized hepatic cords and inflammatory cell infiltration were found in the BDL group, and liver lobules were segmented, leading to the formation of pseudo-lobules. Cellular infiltration and liver injury were significantly reduced in the BDL_RF group. However, there was a slight remission of liver injury in the BDL_GF group compared to the BDL group. Liver fibrosis and collagen depositions in liver tissues were determined by Masson staining (Figure 2B). The Sham group showed normal liver lobular with central veins. The BDL group showed extensive collagen deposition and fibrosis. The fibrotic process was reversed in the BDL_RF group. Fibrosis remained severe in the BDL_GF group, although there appeared to be some improvement compared to the BDL group. All these results suggest that RF exerted a better disease-modifying effect on BDL rats compared to GF.

### RF better alleviates bile duct ligation-induced hepatic oxidative stress than GF

**FIGURE 3:**
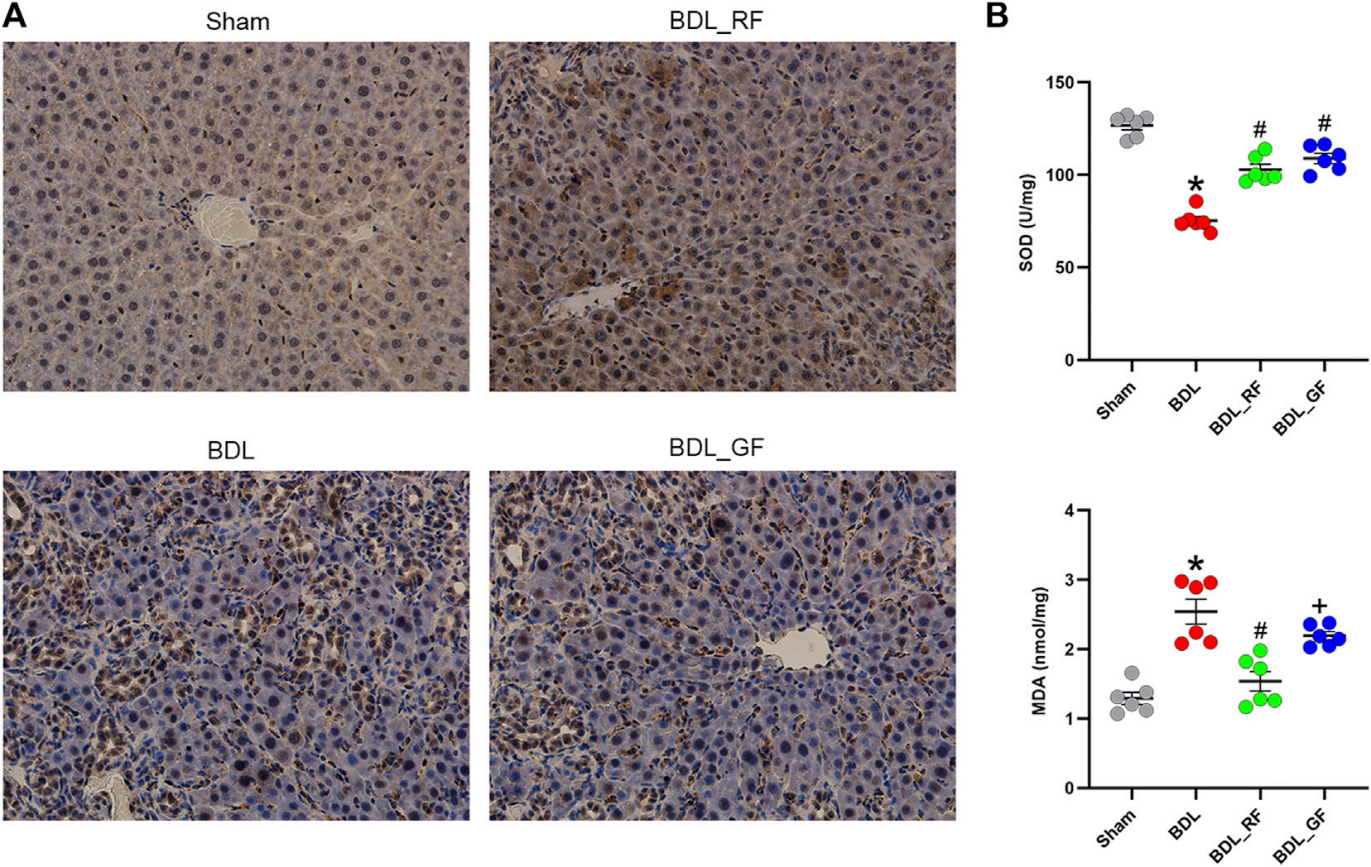
Effects of RF and GF on liver oxidative stress of BDL-rats. **(A)** Representative immunohistochemical staining for NF-E2-related factor 2 (Nrf2) (×400 magnification) of liver tissues. **(B)** Superoxide dismutase (SOD) and malondialdehyde (MDA) levels of liver tissues. (*: *p* < 0.05, compared with the Sham group; #: *p* < 0.05, compared with the BDL group; +: *p* < 0.05, compared with the BDL_RF group, *n* = 6).

Immunohistochemical staining for NF-E2-related factor 2 (Nrf2) of rat livers were shown in Figure 3A. Significantly increased Nrf2-positive areas in BDL and BDL_GF group did not counteract persistent liver damage. BDL_RF group showed both enhanced nuclear localization of Nrf2 and improvement of liver injury. As shown in Figure 3B, the levels of liver superoxide dismutase (SOD) were significantly decreased in BDL group compared to the Sham group. The SOD levels were elevated in both BDL_RF (*p* < 0.05) and BDL_GF (*p* < 0.05) groups compared to the BDL group. Compared with the BDL group, the hepatic malondialdehyde (MDA) levels were attenuated in BDL_RF (*p* < 0.05) group. The MDA levels of BDL_GF group also decreased, but the difference was not significant (*p* > 0.05) compared to the BDL group. The findings indicate that RF better alleviated the oxidative stress damage to the liver of BDL rats.

### RF better prevents bile duct ligation-induced deterioration of serum biochemical indicators than GF

**FIGURE 4:**
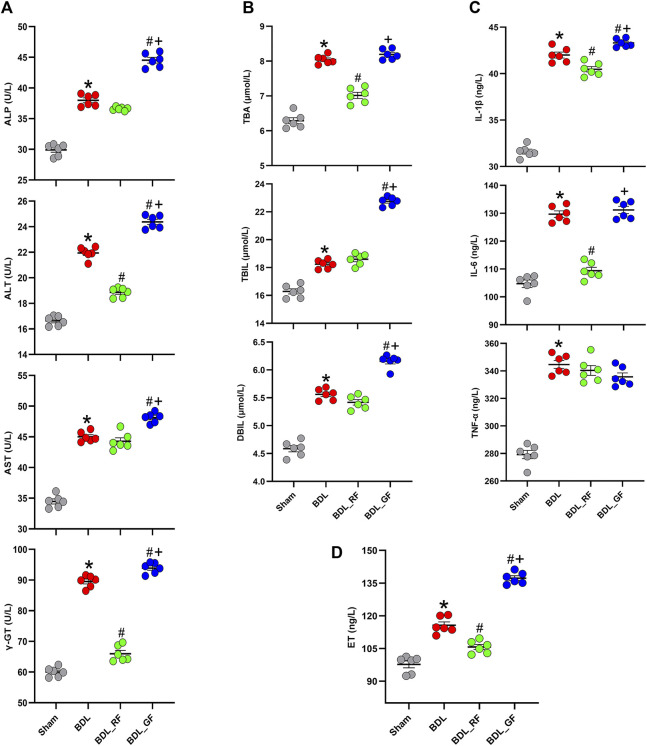
Effects of RF and GF on serum biochemical indicators of BDL-rats. **(A)** Serum levels of alkaline phosphatase (ALP), alanine aminotransferase (ALT), aspartate aminotransferase (AST), and γ-glutamyl transpeptidase (γ-GT). **(B)** Serum levels of total bile acid (TBA), total bilirubin (TBIL), and direct bilirubin (DBIL). **(C)** Serum levels of interleukin-1β (IL-1β), interleukin-6 (IL-6), and tumor necrosis factor-α (TNF-α). **(D)** Serum levels of endotoxin (ET). (*: *p* < 0.05, compared with the Sham group; #: *p* < 0.05, compared with the BDL group; +: *p* < 0.05, compared with the BDL_RF group, *n* = 6).

The biochemical analysis results are presented in Figure 4. Serum levels of 11 indicators were significantly higher in the BDL group compared to the Sham group (*p* < 0.05). RF administration significantly reduced serum levels of alanine aminotransferase (ALT), γ-glutamyl transpeptidase (γ-GT), total bile acid (TBA), interleukin-1β (IL-1β), interleukin-6 (IL-6) and endotoxin (ET) compared to the BDL group (*p* < 0.05). Compared with the BDL group, GF had no significant effect (*p* > 0.05) on serum levels of TBA, IL-6 and tumor necrosis factor-α (TNF-α), and aggravated the deterioration of the other 8 indicators (*p* < 0.05). Almost all indicators (10 out of 11) in the BDL_GF group are higher than those in the BDL_RF group (*p* < 0.05), except for TNF-α (*p* > 0.05). These results show that RF had an ameliorating effect on the biochemical changes induced by BDL, while GF had no effect or made the situation worse.

### RF better ameliorates bile duct ligation-induced enteric metabolic disorder, especially the decrease in bile acids metabolism, compared to GF

**FIGURE 5:**
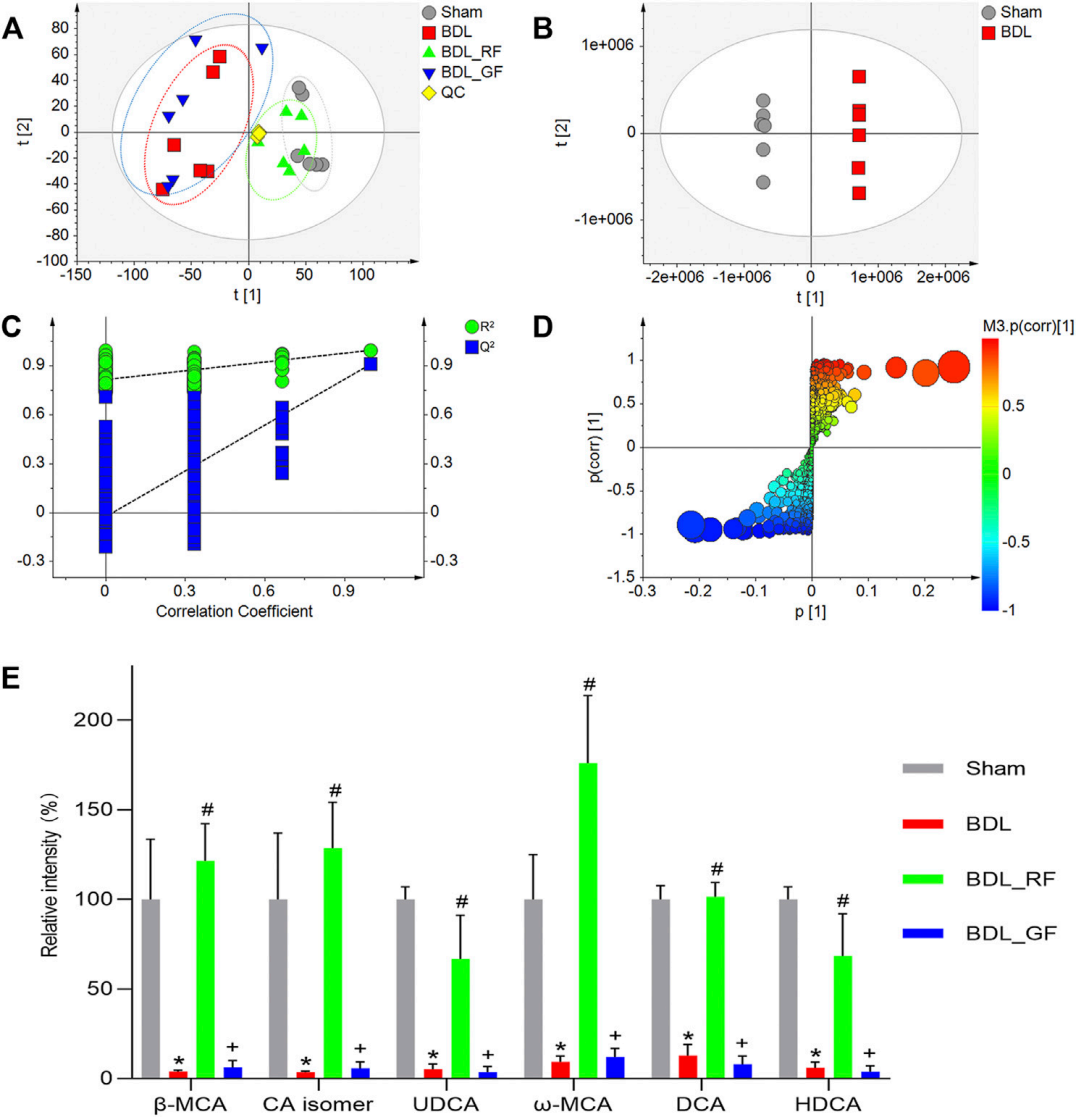
Effects of RF and GF on enteric metabolism of BDL-rats. **(A)** Principal component analysis (PCA) score plot of four groups. **(B)** Orthogonal partial least squares discriminant analysis (OPLS-DA) score plot of the Sham and BDL groups. **(C)** Cross-validation of the constructed model. **(D)** OPLS-DA S-plot of the Sham and BDL groups. The size of each point was shown according to its value of Variable importance on projection (VIP). **(E)** Relative contents variations of bile acid metabolites. (*: *p* < 0.05, compared with the Sham group; #: *p* < 0.05, compared with the BDL group; +: *p* < 0.05, compared with the BDL_RF group, *n* = 6).

The intestinal metabolic differences among groups were characterized using the established UHPLC-Q-Orbitrap MS untargeted metabolomics approach. Quality control (QC) samples were analyzed for the validation of repeatability and stability of analytical methods and instrument. The base peak intensity (BPI) chromatograms were shown in Supplementary Figures S1A,B. All QC samples were within twice standard deviation (SD) in the score map (Supplementary Figure S1C), 92.37% of which variables possessed a relative standard deviation (RSD) less than 30% (Supplementary Figure S1D). The overall intestinal metabolic profile could be visualized in the principal component analysis (PCA) plot (Figure 5A). The tightly clustered QC samples also reflected the stability of the analytical system and high quality of obtained data. Compared with the Sham group, the BDL group showed obvious disorders in enteric metabolism. The BDL_RF group was close to the Sham group. In contrast, the BDL_GF and BDL groups were roughly clustered together.

The orthogonal partial least squares discriminant analysis (OPLS-DA) score plot (Figure 5B) showed two separated clusters between Sham and BDL groups, indicating that BDL led to changes in the enteric endogenous metabolism in rats. After cross-validation, the value of Q^2^ = 0.91 demonstrated this model was robust and not over-fitted (Figure 5C). Metabolites were screened and identified based on S-plot (Figure 5D) and Variable importance on projection (VIP) values (Supplementary Table S1). Metabolites with the value of VIP >5 were defined as the most significant metabolite markers. In particular, totally six bile acid metabolites were discovered, and their identities were temporarily explored by matching retention time (RT), accurate molecular mass (MW), fragment ions with reference substances and the metabolites found in on-line databases. To be specific, the molecular formula of ion at m/z 407.2802 (RT 8.70) was calculated as C_24_H_39_O_5_ with mass accuracy less than 5 ppm, which corresponded to the deprotonated molecular ions [M-H]^-^ of β-Muricholic acid (β-MCA). Ion (RT 8.70) at m/z 453.2862 had 46 Da more than that of [M-H]^−^ of β-MCA, which could be the ion [M + HCOO]^−^ of β-MCA. The ions at m/z 391.2853 (RT 10.54) and m/z 437.2911 (RT 10.54) were assigned as [M-H]^−^ and [M + HCOO]^−^ of Ursodeoxycholic acid (UDCA). The ions at m/z 407.2803 (RT 8.15), m/z 453.2863 (RT 8.15), and m/z 815.5684 (RT 8.15), could be respectively [M-H]^−^, [M + HCOO]^−^, and [2M-H]^−^ of ω-Muricholic acid (ω-MCA). Similarly, the ions at m/z 391.2858 (RT 10.56), m/z 437.2912 (RT 10.56), and m/z 783.5783 (RT 10.56), could be respectively [M-H]^−^, [M + HCOO]^−^, and [2M-H]^−^ of Hyodeoxycholic acid (HDCA). Ions at RT 8.77 and RT 15.02 were identified to be an isomer of Cholic acid (CA isomer) and Deoxycholic acid (DCA). Their related parameters are listed in Supplementary Table S1. Furthermore, the box plots of relative contents of the six fecal BAs were demonstrated in Figure 5E. Compared with the Sham group, the relative intensities of six fecal BAs, including three primary BAs (β-MCA, CA isomer, UDCA) and three secondary BAs (ω-MCA, DCA, HDCA) in the BDL group, were significantly decreased (*p* < 0.05). All six BAs were significantly increased in the BDL_RF group compared with the BDL group (*p* < 0.05). However, there was no significant difference between BDL_GF group and BDL group.

### For the overall profile of the gut microbiota, RF and GF have both similar and different effects on bile duct ligation-rats

**FIGURE 6:**
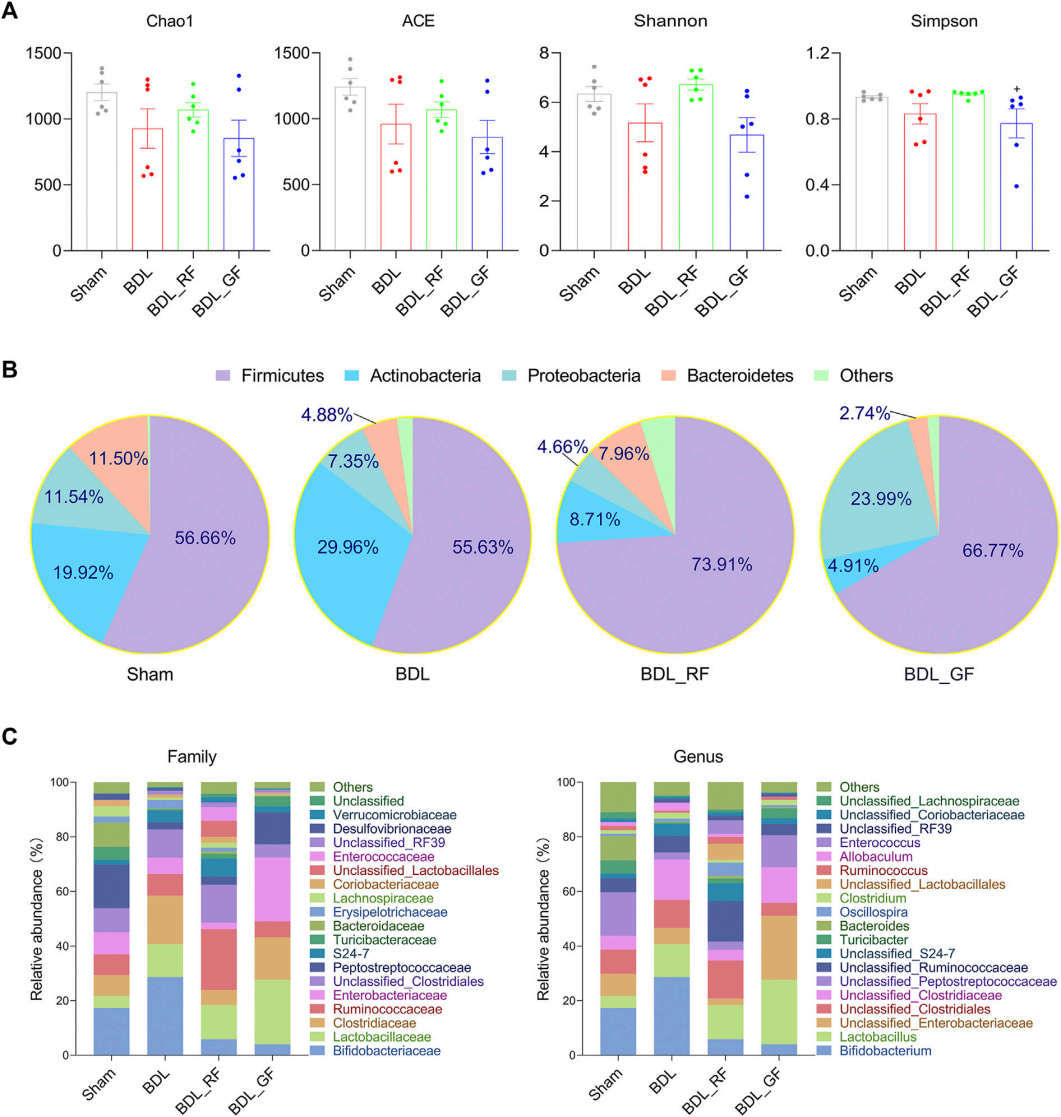
Comparison of gut microbiota profile between the four groups. **(A)** The Chao1 and abundance-based coverage estimator (ACE), as well as the Shannon and Simpson index. (+: *p* < 0.05, GF_BDL compared with the RF_BDL group, *n* = 6). **(B)** Phylum level microbiota composition. Data are the means, mainly showing the top 5 species in relative abundance. **(C)** Family and genus level microbiota composition. Data are the means, mainly showing the top 20 species in relative abundance.

The Chao1 and abundance-based coverage estimator (ACE) mainly reflect the richness of species. While the Shannon and Simpson index, mainly reflect the diversity of species. As shown in Figure 6A, the Chao1 and ACE, as well as the Shannon and Simpson index, decreased in the BDL group. The four indices were even lower in the BDL_GF group than in the BDL group. The four indices in the BDL_RF group showed recovery tendency. However, none of these differences were significant (*p* > 0.05). The Simpson index was higher in the RF group than in the GF group (*p* < 0.05). As shown in Figure 6B, at the phylum level, the four groups differed in their major microbiota composition. There were some similarities between BDL_RF and BDL_GF groups, such as higher abundance of Firmicutes and lower abundance of Actinobacteria. The abundance of Proteobacteria was lower in the BDL_RF group and higher in the BDL_GF group. In addition, BDL decreased the abundance of the Bacteroidetes. RF treatment upregulated its abundance, and GF treatment made it even lower. These in turn reflect the different effects of RF and GF on the intestinal bacteria of BDL rats. As shown in Figure 6C, at the family and genus level, the four groups differed in the composition of the major species. The results in this section mainly reflect the different trends in the overall profile of gut microbiota between groups. The effect of BDL on important bacteria and the interventional role of RF and GF, as well as the relationship of these bacteria with BAs metabolism and disease progression will be focused on in the next section.

### RF has superior effects than GF on the regulation of bacteria potentially associated with bile acids metabolism and disease progression in bile duct ligation-rats

**FIGURE 7:**
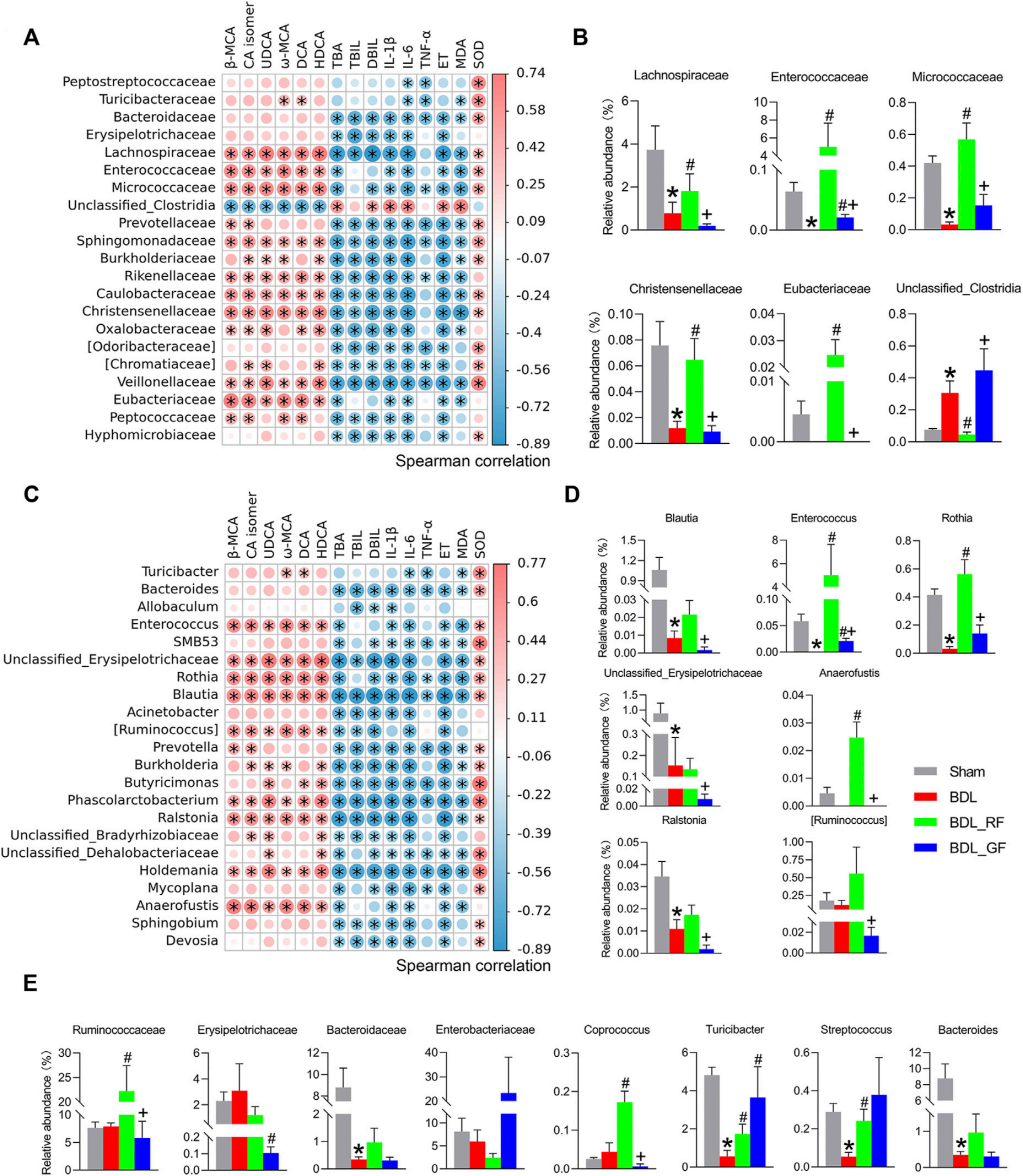
Bacteria potentially associated with bile acids metabolism and disease progression. **(A**,**C)** Heatmaps of Spearman correlations between bacteria and enteric bile acids, serum biochemical indicators, and liver oxidative stress at the family and genus levels. The heatmap was created based on data on bacterial abundance and enteric bile acids, serum biochemical indicators, and liver oxidative stress levels. A Spearman correlation coefficient close to 1 (>0.6) indicates a strong positive correlation, while a Spearman correlation coefficient close to −1 (<−0.6) indicates a strong negative correlation. (*: *p* < 0.05, *n* = 6). **(B**,**D)** Bacteria with intergroup differences in relative abundance among those screened by Spearman correlation analysis at the family and genus levels. **(E)** Intergroup differences in the relative abundance of other important bacteria at the family and genus levels. (*: *p* < 0.05, compared with the Sham group; #: *p* < 0.05, compared with the BDL group; +: *p* < 0.05, compared with the BDL_RF group, *n* = 6).

After correlation analysis and between-group difference analysis based on the results of the correlation analysis, a total of six families and seven genera were screened for close association with BAs metabolism and disease progression (Figures 7A–D). As shown in Figure 7A, for instance, the relative abundance of Lachnospiraceae was positively correlated with the levels of six enteric BAs and the hepatic antioxidant index SOD, and negatively correlated with three serum BA-related indicators, two serum inflammatory indexes, ET, and the hepatic oxidative stress index MDA. This implies that the increase of Lachnospiraceae may predict some recovery of BAs metabolism and improvement in the condition of BDL-rats. Meanwhile, the relative abundance of Lachnospiraceae was significantly different among the four groups. Compared with the Sham group, BDL obviously reduced the abundance of Lachnospiraceae. The abundance of Lachnospiraceae was higher in RF_BDL group (*p* < 0.05) but lower in GF_BDL group (*p* > 0.05), compared to the BDL group. The relative abundance of Lachnospiraceae in RF_BDL group was significantly higher than that in GF_BDL group (*p* < 0.05, Figure 7B). Similarly, the relative abundance of *Enterococcaceae*, *Micrococcaceae*, *Christensenellaceae*, and *Eubacteriaceae* that decreased in the BDL group were significantly higher in RF_BDL group (*p* < 0.05) than in GF_BDL group (Figure 7B). In contrast, BDL increased the relative abundance of Unclassified_Clostridia, compared to the Sham group. The relative abundance of Unclassified Clostridia was lower in RF_BDL group (*p* < 0.05) but higher in GF_BDL group (*p* > 0.05), compared to the BDL group (Figure 7B). The relative abundance of Unclassified_Clostridia in RF_BDL group was significantly lower than that in GF_BDL group (*p* < 0.05, Figure 7B). At the genus level, the relative abundance of *Blautia*, *Enterococcus*, *Rothia*, Unclassified_*Erysipelotrichaceae*, *Anaerofustis*, *Ralstonia*, and [*Ruminococcus*] that declined in the BDL group were significantly higher in RF_BDL group (*p* < 0.05) than in GF_BDL group (Figure 7D). In addition, intergroup comparisons of eight other important bacteria (*Ruminococcaceae*, *Erysipelotrichaceae*, *Bacteroidaceae*, *Enterobacteriaceae*, *Coprococcus*, *Turicibacter*, *Streptococcus*, and *Bacteroides*) are also presented in Figure 7E. These results indicate that RF better restored bacteria that potentially involved in BAs metabolism and disease progression in BDL-rats compared to GF.

## Discussion

In this study, a BDL model was used to evaluate two different harvesting periods of FF (RF & GF). The results showed that BDL caused a series of pathological, biochemical, enteric metabolic and gut microbiota changes in rats, and that RF was superior to GF in reversing these changes. We speculate that these changes should be interrelated, and that BAs and gut microbiota may be important and critical links between them. The differences in the effects of RF and GF on BDL rats and the mechanisms responsible for such differences are discussed as follows.

BDL causes hepatic and intestinal impairment and loss of appetite in experimental animals. In the present study, RF restored the normal trend of weight gain in BDL rats, which was in contrast to the continuous weight loss caused by GF. The repair of liver tissue and attenuation of cellular infiltration by RF in BDL rats were observed. RF also alleviated collagen deposition and fibrosis in the liver caused by BDL. The effect of RF in restoring body weight in BDL rats may be partly related to its improvement in liver physiological function.

Impaired hepatic bile flow induced by BDL can lead to excessive accumulation of toxic BAs in hepatocytes, causing hepatic cholestasis and liver injury. Nrf2 is a regulator of hepatic detoxification and antioxidant mechanisms. Therefore, the activation of Nrf2 is considered to be useful for prevention or treatment of cholestatic liver injury (Shin et al., 2013). The activated Nrf2 in BDL group confirmed the spontaneous response to oxidative stress. The beneficial antioxidant effects of FF through activation of Nrf2 have been recently found in animal models of inflammatory liver injury (Zhao et al., 2017) and melanoma (Bao et al., 2016). In this study, RF and GF elevated the levels of SOD, one of the Nrf2 targets in liver. However, RF and GF have different effects on nuclear localization of Nrf2, MDA, and liver injury. Nrf2 half-life is around 13–20 min. In oxidative stress and in the presence of antioxidant molecules, the half-life of Nrf2 is duplicated (Dinkova-Kostova et al., 2001). Thus, we speculate that RF may have more antioxidant components entering the circulation and liver than GF, resulting in a longer half-life and enhanced nuclear localization of Nrf2, which in turn exerts better antioxidant and hepatoprotective effects. Additional experiments are warranted.

The increase in serum transaminases and TBA, TBIL, DBIL caused by BDL seems to reflect the toxic effects of regurgitated BAs to the liver. For these indicators of liver function and bile acid circulation, RF provided some improvement, while GF made the situation worse. The significant difference in the effect of RF and GF on these indicators suggests that they do not behave consistently in response to hepatocellular injury (reflected by ALT and AST), biliary epithelial cell injury (reflected by ALP and γ-GT) and bile acid imbalance (reflected by TBA, TBIL and DBIL) caused by BDL. The liver, located at the gateway of the portal blood flow draining the gastrointestinal tract, is strategically and uniquely positioned as the final barrier to prevent gut bacteria and bacterial products, such as ET, from entering the systemic blood stream. Therefore, the increase in serum ET caused by BDL indicates not only a deficiency of the hepatic barrier function, but also implies a disturbance of the gut microbiota. Similar to the aforementioned indicators, RF reduced ET levels, while GF exacerbates the ET elevation. It has been reported that IL-6 is the best marker of tissue inflammation and injury (Hu et al., 2020). Due to ET stimulation, Kupffer cells secrete a large number of active mediators, including cytokines such as IL-1β, IL-6, TNF-α, and reactive oxygen species (ROS). This also results in elevated hepatic function markers, eventually triggering the liver damage (Sato et al., 2016; Xie et al., 2019; Zou et al., 2020). In this study, serum IL-1β and IL-6 were increased in BDL rats and were reduced by RF. However, GF failed to bring down these cytokines. Overall, in our study, cholestasis caused by BDL left the rats in a worse condition, as evidenced by lower body weight, structural damage to liver tissue and impaired liver function. For these pathological changes, the beneficial effects of RF, rather than GF, may be generated by reducing ET, hepatic oxidative stress and systemic inflammation.

As a result of BDL, the natural flux of BAs from the liver to the gut is impaired, causing not only an accumulation of toxic BAs in the liver, but also a deficiency of BAs in the intestine (Alaish et al., 2013). Therefore, on the one hand, bacterial overgrowth and increased intestinal permeability occur in BDL due to the decreased antibacterial and intestinal protective function of BAs (De Minicis et al., 2014). This promotes the translocation of bacteria and bacterial products, such as ET, from the permeable gut into the liver via the porta circulation, exacerbating hepatic inflammation and fibrosis (Konturek et al., 2018). On the flip side, BAs and other enteric metabolites are also modified and transformed by intestinal bacteria. Thus, the interaction between these metabolites and gut microbiota links the intestine closely to the liver and plays a key role in the cholestatic disease process (Schnabl, 2013). Changes in enteric metabolites and intestinal bacteria caused by BDL, as well as the effects of RF and GF administration were designed in the study. Metabolomics analysis showed that RF ameliorated the overall metabolic disorder caused by BDL in rats. However, GF had no significant effect. In particular, BDL induced a significant decrease in the level of major enteric BAs. This indicates that BDL intercepts the bile flow secreted from the liver to the intestine. In other words, reduced levels of bile acid-related metabolites may also be primarily involved in shaping the enteric metabolic profile in the pathological state of BDL-induced cholestasis. To some extent, fecal BAs reflect both the level of intestinal BAs and the amount of BAs excreted through the stool. In rodents, β-MCA is the main primary BA, while ω-MCA and HDCA account for the most in secondary BAs (de Aguiar Vallim et al., 2013; Li and Chiang, 2015; Lin et al., 2020). In terms of the BA pool, elevated enteric BAs (β-MCA, CA isomer, UDCA, ω-MCA, DCA, HDCA) after RF intervention in BDL rats could promote the excretion of BAs by fecal loss. It would also reasonably be expected to result in a relaxation of circulating toxic BAs accumulation. From the perspective of the intestine itself, increased levels of enteric bile acids will play a beneficial role in protecting resident bacteria, inhibiting opportunistic pathogenic bacteria, and restoring the gut microecology.

The metabolism of primary and secondary BAs in the gut is regulated by the enzymes of specific bacteria. This creates an interactive relationship between BAs and intestinal bacteria. It is worth exploring whether the alteration of BAs by BDL is accompanied by an alteration of intestinal bacteria. It is also intriguing whether the restorative effect of RF on BA disorders is associated with its effect on intestinal bacteria. Of course, it is especially crucial to clarify how the improvement of BAs metabolism and the regulation of gut microbiota contribute to the recovery of the pathological condition caused by BDL. Unlike GF, RF showed a tendency to elevate the reduction in flora richness and diversity caused by BDL. At the phylum level, GF elevated the average abundance of Proteobacteria, which contains many pathogens. While, RF and GF increased and decreased the average abundance of Bacteroidetes, which is closely related to BAs metabolism, respectively. At the family and genus level, the present study also showed that RF and GF interventions had different effects on the altered flora composition due to BDL. However, these results only reflect different trends in the overall profile of the microbiota.

To investigate whether the effects of BDL, RF and GF on gut microbiota were associated with BAs metabolism and disease progression, Spearman’s correlation analysis and between-group difference analysis were successively performed. The results indicated that Lachnospiraceae, for instance, was related to increased levels of major fecal BAs and decreased levels of three serum bile acid-related indicators. Dysregulation of BAs metabolism is a key hallmark of cholestatic disease. A shrinking enteric BA leads to increases in microbes with potent pro-inflammatory molecules coupled with harmful metabolites such as ET, which in turn add to the burden of liver damage (Ridlon et al., 2013). The recovery of BAs metabolism means, to some extent, the improvement of cholestasis. In the present study, we also observed that Lachnospiraceae was associated with an increase in hepatic antioxidant marker and a decrease in hepatic oxidative stress level, two serum inflammatory markers and ET level. This implies that the increase of Lachnospiraceae may predict enhancement of BAs metabolism and remission in the condition of BDL rats. Thus, Lachnospiraceae may be a beneficial family for the improvement of BDL-induced cholestatic disease. BA formation is mediated exclusively by gut microbiota via deconjugation (the first step of BAs metabolism, removal of glycine or taurine from conjugated BAs to obtain primary BAs), dehydroxylation (the second step of BAs metabolism, removal of a hydroxyl group from primary BAs to produce secondary BAs) and other reactions. The dehydroxylation appears restricted to a limited number of intestinal anaerobes in the order of Clostridiales, including Lachnospiraceae, Ruminococcaceae and Blautia (Ridlon et al., 2006; Ridlon et al., 2013). Lachnospiraceae and Blautia are also ranked among the bacterial strains hold bile salt hydrolase (BSH) genes, catalyzing the deconjugation transformation (Song et al., 2019). These findings suggest the usefulness of Lachnospiraceae for the critical two-step conversion of intestinal BAs. Therefore, our results indicate that promoting the production of intestinal BAs and excretion of fecal BAs by upregulating the abundance of bacteria such as Lachnospiraceae may be one of the mechanisms by which RF alleviated cholestasis in BDL rats. It is worth noting that Lachnospiraceae and Ruminococcaceae are also butyrate producing bacteria. Butyrate, as a representative short-chain fatty acid (SCFA), has various protective effects on the innate and adaptive immune systems, increasing anti-bacterial peptides and suppressing inflammatory cytokine (Qin et al., 2014; Fukui, 2017). In short, these RF-boosted bacteria, which promote BAs metabolism and maintain intestinal health, can be defined as “good” bacteria for BDL-induced cholestasis.

The other four families obtained from the screening, *Enterococcaceae*, *Micrococcaceae*, *Christensenellaceae*, and *Eubacteriaceae*, were affected by BDL, RF, and GF in a similar way to *Lachnospiraceae*. Among them, *Enterococcaceae* is a family rich in strains with BSH activity (Song et al., 2019). *Micrococcaceae* (Shao et al., 2020) and *Eubacteriaceae* (Nagano and Yano, 2020) are SCFAs producing bacteria. *Christensenellaceae*, a family that is highly heritable and shows compelling associations with host health, appeared to be depleted in conditions associated with inflammation (Waters and Ley, 2019). Unclassified_*Clostridia* is the only possible “bad” family in the screened species. Its relative abundance was elevated by BDL and GF and pulled down by RF. In a previous study, an increase of *Clostridium* was found in mice at day 7 after BDL. They postulated that increased abundance of *Clostridium* may contribute to the overall progression of liver disease by promoting Lipopolysaccharide (LPS) induced liver injury and inflammation (Cabrera-Rubio et al., 2019). Since no definite family or genus has been obtained, further work on bacterial identification and functional exploration is needed. At the genus level, *Blautia*, *Enterococcus* and *Rothia*, belong to the first three families screened at the family level, *Lachnospiraceae*, *Enterococcaceae* and *Micrococcaceae*, respectively. These genera and the families to which they belong are essentially similar in function and intergroup variation trends. The role of *Blautia* has been previously described (Ridlon et al., 2006; Ridlon et al., 2013; Song et al., 2019), while the latter two are both overwhelmingly dominant species in their respective families. Consistent with these three bacteria, the relative abundances of the other four genera obtained from the screening, i.e., Unclassified_*Erysipelotrichaceae*, *Anaerofustis*, *Ralstonia* and [*Ruminococcus*], were all significantly higher in the RF group than in the GF group. A study of Crohn’s disease found *Erysipelotrichaceae* among the predominant taxa within the secondary BA-dominant assemblages (Connors et al., 2020). *Anaerofustis*, a fibrolytic bacteria, could be involved in the fermentation of carbohydrates and glucose metabolism. A study of inflammatory bowel disease (IBD) identified an enrichment of *Anaerofustis* in the control group (Masoodi et al., 2020). The decreased body weight in the BDL and GF groups in our study may be related to the depletion of such digestive bacteria in the pathological state. *Ralstonia* is the best studied genus with respect to polyhydroxyalkanoates (PHAs) accumulation (Pötter et al., 2002). PHAs, the polymers of β-hydroxy SCFAs, can be degraded in the gastrointestinal tract of mammals and result in SCFAs release. Therefore, PHAs could beneficially affect the host–microbe interaction in the gut in a way similar to SCFAs (Defoirdt et al., 2009). *Ruminococcus* species with BSH, oxidation and epimerization activities are extensively involved in BAs metabolism and have beneficial effects in promoting host intestinal homeostasis (Chen et al., 2018; Jia et al., 2018; Connors et al., 2020; Vega-Magaña et al., 2020). The different effects of RF and GF on these bacteria imply that, in addition to BA-metabolizing bacteria, the improvement of RF in BDL rats is also related to its ability to promote the growth of other beneficial and protective bacteria in the intestine.

There are species that did not stand out in the two-step screening. One of the reasons could be their discrete data. However, considering their important functions found in previous studies and their close relationship with the bacteria obtained from the screening in this study, their intergroup differences were also analyzed and shown. *Ruminococcaceae* and *Coprococcus*, similar to *Lachnospiraceae*, are both BA-dehydroxylating and SCFAs producing bacteria (Ridlon et al., 2013; Qin et al., 2014; Fukui, 2017; Connors et al., 2020). The positive relationship of *Erysipelotrichaceae* with secondary BAs has been described (Connors et al., 2020). Studies have also found a positive correlation between Turicibacter and both secondary and primary BAs (Chen et al., 2018; Kemis et al., 2019). In addition, several minor secondary BAs were positively associated with BSH producing genera *Streptococcus* (Ridlon et al., 2013; Chen et al., 2018; Huang et al., 2019; Connors et al., 2020). *Bacteroidaceae* and *Enterobacteriaceae* are two representative families with high relative abundance. The prevalence of BSH activity in *Bacteroidaceae* species reflects the wide distribution of BSH genes in gut microbiota. This is quite different from the narrow distribution of bacteria that catalyze the production of secondary BAs. In other words, for the conversion of conjugated BAs to primary BAs, these BSH producers are interchangeable with each other (Ridlon et al., 2006; Song et al., 2019). As demonstrated in Figure 7, even RF failed to have a remedy for the decline in Bacteroidaceae (including its major genus *Bacteroides*) caused by BDL. In contrast, *Enterococcaceae* (including its major genus *Enterococcus*), which also has a large number of BSH strains, improved substantially after RF administration and surpassed the Sham group. Almost all strains in *Bacteroides* and *Enterococcus* contain BSH protein sequences (Song et al., 2019). When considered in terms of BAs metabolic homeostasis, we speculated that this could be an alternative compensatory measure taken by the organism through intestinal bacteria under bile restriction stress. In pathological conditions, this potential defense mechanism requires pharmacological intervention to be amplified and realized. It remains unknown what the other consequences of the RF-induced increase in the alternative bacteria will be, other than our concern about the restoration of BAs metabolism. The roles and exact mechanisms involved are fascinating. *Enterobacteriaceae*, on the other hand, contains a large number of conditional pathogenic and pro-inflammatory members. Previous studies have shown that gut microbiome changes or dysbiosis in patients with chronic liver disease are often accompanied by a decrease in BA-dehydroxylating bacteria and an increase in pathogenic Gram-negative bacteria, particularly *Enterobacteriaceae* (Shao et al., 2021). In a study on hepatic encephalopathy, a positive correlation was found between *Enterobacteriaceae*, endotoxemia inflammation and oxidative stress indicators (Ridlon et al., 2013). The expansion of *Enterobacteriaceae* is hypothesized to initiate or exacerbate the overactive immune response and the ensuing tissue damage characteristic of disease (Connors et al., 2020). In the current study, *Enterobacteriaceae*, referred to as LPS releasing and ET producing bacteria, tended to be enriched in the GF group. This trend is consistent with the previously mentioned case of Proteobacteria to which it belongs. These “bad” bacteria may worsen the vicious cycle characterized by gut dysbiosis, metabolic imbalance and organ injuries, resulting in a poor prognosis of GF administration.

The above results of BDL modeling, FF intervention, difference comparisons, and stepwise screening in our study indicate that the dynamic interactions between intestinal BAs metabolism and gut microbiota were closely associated with disease changes. A picture is now starting to emerge regarding the liver-BA-microbiome-gut axis in BDL-induced cholestatic disease. BDL caused a decrease in both intestinal primary and secondary BA levels, accompanied by depletion of the associated BA-metabolizing bacteria. These changes are firstly a consequence of the disease and secondly act as irritants to further aggravate the severity of the disease. In addition, protective SCFAs producing bacteria and destructive ET producing bacteria may also be involved in disease progression. Some of the bacteria obtained from screening that appear to be very beneficial for disease recovery combine two types of functions: intestinal BAs metabolism and gut protection. The restorative effect of RF may be due precisely to the capture of these so-called key bacteria, which accelerate BAs metabolism and enhance intestinal protection by adjusting these core bacteria to detoxify and alleviate the disease. We focused specifically on two groups of BA-metabolizing bacteria: secondary BA producing bacteria and primary BA producing bacteria. The former group of bacteria, which has a narrow distribution and thus a relatively more important role, is at greater risk of depletion after being affected by disease. This type of bacteria was better recovered by RF. For the latter group of bacteria, which is widely distributed, RF responded mainly through alternative bacteria. GF did not show similar effects and had a tendency to encourage the reproduction of certain conditionally pathogenic bacteria.

**FIGURE 8:**
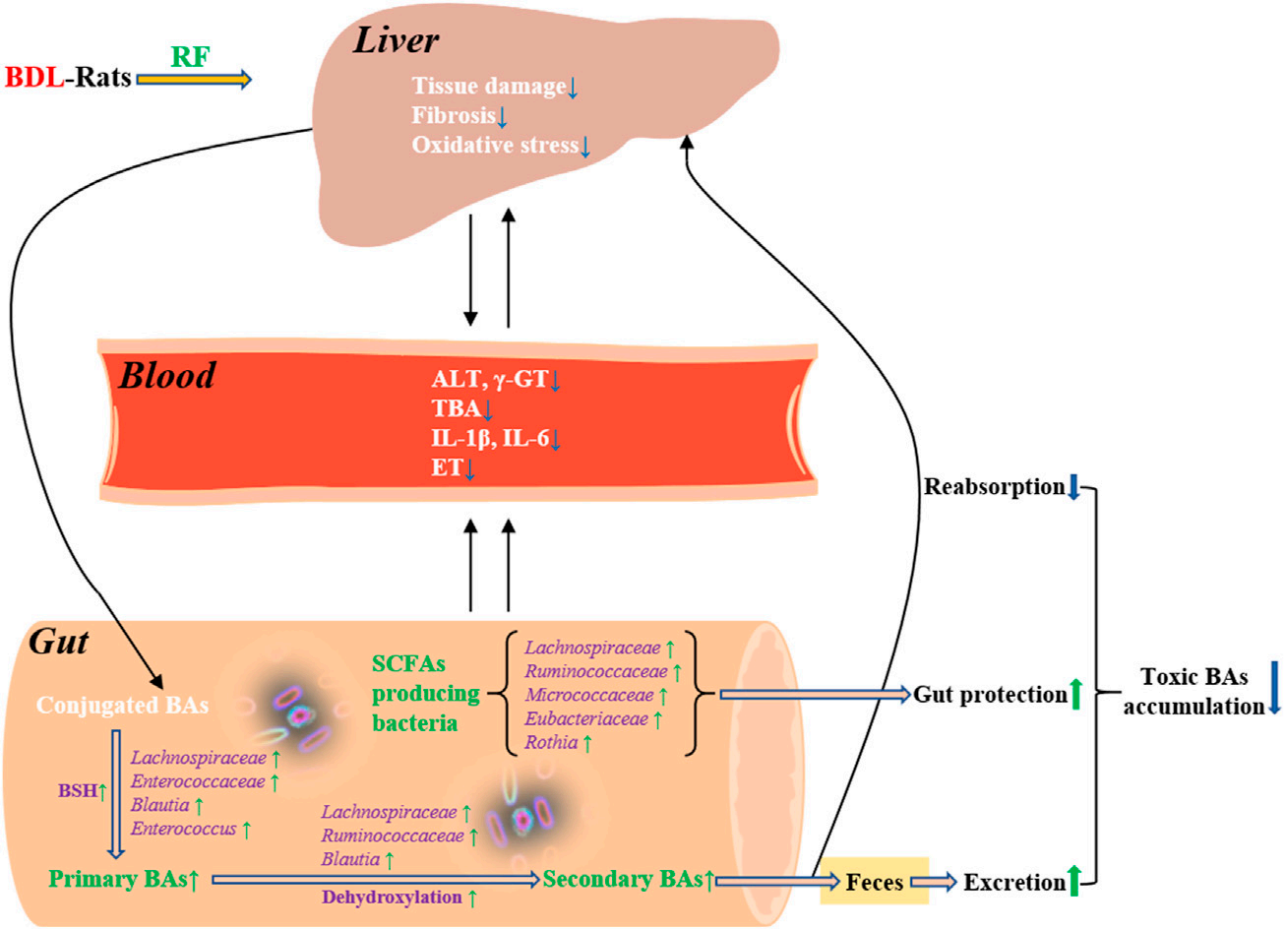
Detoxifying effects of RF on BDL-rats and the potential mechanisms involving bile acids metabolism and gut microbiota.

Cholestasis is a pathologic condition characterized by impairment or cessation of the bile flow with consequent liver damage. Accumulation of toxic BAs is central to the pathogenesis of cholestatic disease. Therefore, reducing BAs overload is a therapeutic goal for the management of cholestasis. This goal could be achieved by strategies such as inhibition of BAs reabsorption from intestine. Recent studies suggest that gut microbiota play an important role in the pathophysiology of cholestatic disease and targeting microbiota may offer novel treatment options for this disease (Liu et al., 2020). In the present study, administration of RF increased the abundance of primary BA producing bacteria and secondary BA producing bacteria of BDL-rats. Their deconjugating and dehydroxylating effects accelerated fecal excretion of BAs, because deconjugated and dehydroxylated BAs are less hydrophilic and therefore are less likely to be reabsorbed. To some extent, more fecal BAs excretion relieved the body from toxic BAs accumulation. The putative detoxifying effects and potential mechanisms of RF on BDL-rats are illustrated in Figure 8.

As a well-known herb for clearing heat and detoxifying, the heat-clearing effects of FF have been studied extensively. These studies seem to show that GF has a stronger heat-clearing effect than RF (Nishibe, 2002; Dong et al., 2017; Wang et al., 2018). FF is the main ingredient of Forsythiae honeysuckle (Lian-Hua-Qing-Wen) prescription recommended in the TCM treatment protocol of COVID-19 in China (Liang et al., 2021). The better heat-clearing ability of GF (mainly based on the anti-inflammatory properties) might explain the more frequent uses of GF in these TCM prescriptions (Nishibe, 2002; Dong et al., 2017; Wang et al., 2018). However, in this study, the detoxifying effect on BDL-induced accumulation of toxic BAs appeared to be better in RF than in GF. We assume that the direct and strong anti-inflammatory and anti-pathogenic effects of GF may be suitable for clearing heat against Fei (roughly lung and respiratory tract, Upper Jiao as referred to in TCM theory) infections. Nevertheless, severe heat-clearing effect of GF may cause deficiency and coldness of the Pi (roughly corresponds to the spleen) and Wei (roughly corresponds to the stomach), when used in middle and lower Jiao and result in poor prognosis. In this study, the weakness of the body such as loss of appetite, indigestion, disruption of normal enterohepatic circulation, and imbalance of gut microbiota caused by BDL may amplify the bitter, cold, and violent characteristics of GF. This could become a disadvantage of GF and be detrimental to treatment. While the indirect and gentle gut microbiota regulating and detoxifying effects of RF are more useful for the conveyance and dispersion treatment of hepatobiliary diseases. Corresponding in-depth experiments need to be designed and conducted in the future.

In fact, the use of FF for hepatobiliary diseases is also available in ancient TCM books. Zhang Zhong-Jing’s herbal prescriptions are considered to be the classic and are still used as the guidelines for TCM clinical work nowadays. In Shang-Han Lun (Treatise on Exogenous Febrile Diseases, written around 210 AD in the Han Dynasty), he recorded the treatment of jaundice with Ma-Huang Lian-Qiao Chi-Xiao-Dou Tang (Ephedra Forsythiae Adzuki-bean decoction) (Xiong, 2000). However, since ancient times, it has not been conclusive whether GF or RF should be used for this prescription. Our research suggests that RF may be a good choice. In terms of TCM theory, RF is also closer to the medicinal properties and odor of Forsythiae root as chosen in the original Shang-Han Lun. This seems to be a remote echo and mutual corroboration of ancient TCM experience and modern pharmacological experiments.

The intestinal tract is the primary site of interaction between bile acid metabolites and bacteria. The interaction between the two profoundly affects the host, and the physio-pathological state of the host in turn affects the intestinal environment. This triangular relationship is very interesting in hepatobiliary diseases, reflecting the fact that the disease progression is not a single, static but a multiple and dynamic process. The host, flora, as well as their co-metabolites, are equally indispensable. This holistic concept is similar to the view of TCM, according to which treatment with herbs or combinations of herbs does not act on a single target, but on a multi-actor collection. The elements of this collection are always interacting and dynamically changing in a multi-dimensional space-time.

## Conclusion

In the face of BDL-induced cholestasis, RF and GF showed different effects. BAs and gut microbiota may be the main players in this process and are essential for improvement of the condition, but should not be the whole picture. The causes and progression of cholestasis are complex. In addition to toxic BAs, pathologically elevated and accumulated other endogenous substances may also cause toxic damage to the organism. In-depth studies to more comprehensively assess the detoxifying effects of FF (RF and GF) and explore the intrinsic mechanisms will open up new perspectives for the medicinal research of FF.

At the end, we are aware of the limitations of the present study. Firstly, the pathological manifestations of the animals were related to the duration of the BDL model. A long period of time may result in more deaths in the model groups of animals, whereas if not enough time has elapsed, changes in the flora have not yet occurred significantly. We chose 3 weeks based on our pre-experimental experience, therefore, the time factor cannot be ignored when comparing with other relevant BDL studies. Second of all, the administration of FF in this study was converted from the recommended oral dose in humans, and the high, medium and low doses were not designed for the time being. Finally, this paper focuses on the possibility that FF (RF) may alleviate cholestasis by modulating the gut microbiota to alter the BA profile to reduce its reabsorption and increase its excretion. It is important to note that the BA profile is influenced by *de novo* BA synthesis and the feedback regulatory pathway. The complex interactions that occur between nuclear receptors, G protein-coupled receptors and BA molecules in the pathway (de Aguiar Vallim et al., 2013; Liu et al., 2020; Shao et al., 2021) are not addressed at this time. Whether FF acts directly or indirectly on these enzymes of BA synthesis, receptors, and relevant transporters is a work in progress and is expected to follow.

To our knowledge, there are few studies comparing the *in vivo* detoxifying effects of the two types of FF, of which those involving the gut microbiota have not been reported. Such comparative studies are urgently needed and have strong theoretical and practical implications. We hope this is a promising exploration and a good start.

## Data Availability

The datasets presented in this study can be found in online repositories. The names of the repository/repositories and accession number(s) can be found below: https://www.ncbi.nlm.nih.gov/, PRJNA837391.
