# Supplementary material for: Better detoxifying effect of ripe forsythiae fructus over green forsythiae fructus and the potential mechanisms involving bile acids metabolism and gut microbiota
Source: Front Pharmacol. 2022 Aug 12;13:987695. doi: 10.3389/fphar.2022.987695 (PMC9417252; doi:10.3389/fphar.2022.987695)
Supplement: Supplementary file 1 [file DataSheet1.docx]

Supplementary Material

# Supplementary Figures and Tables

## Supplementary Figures


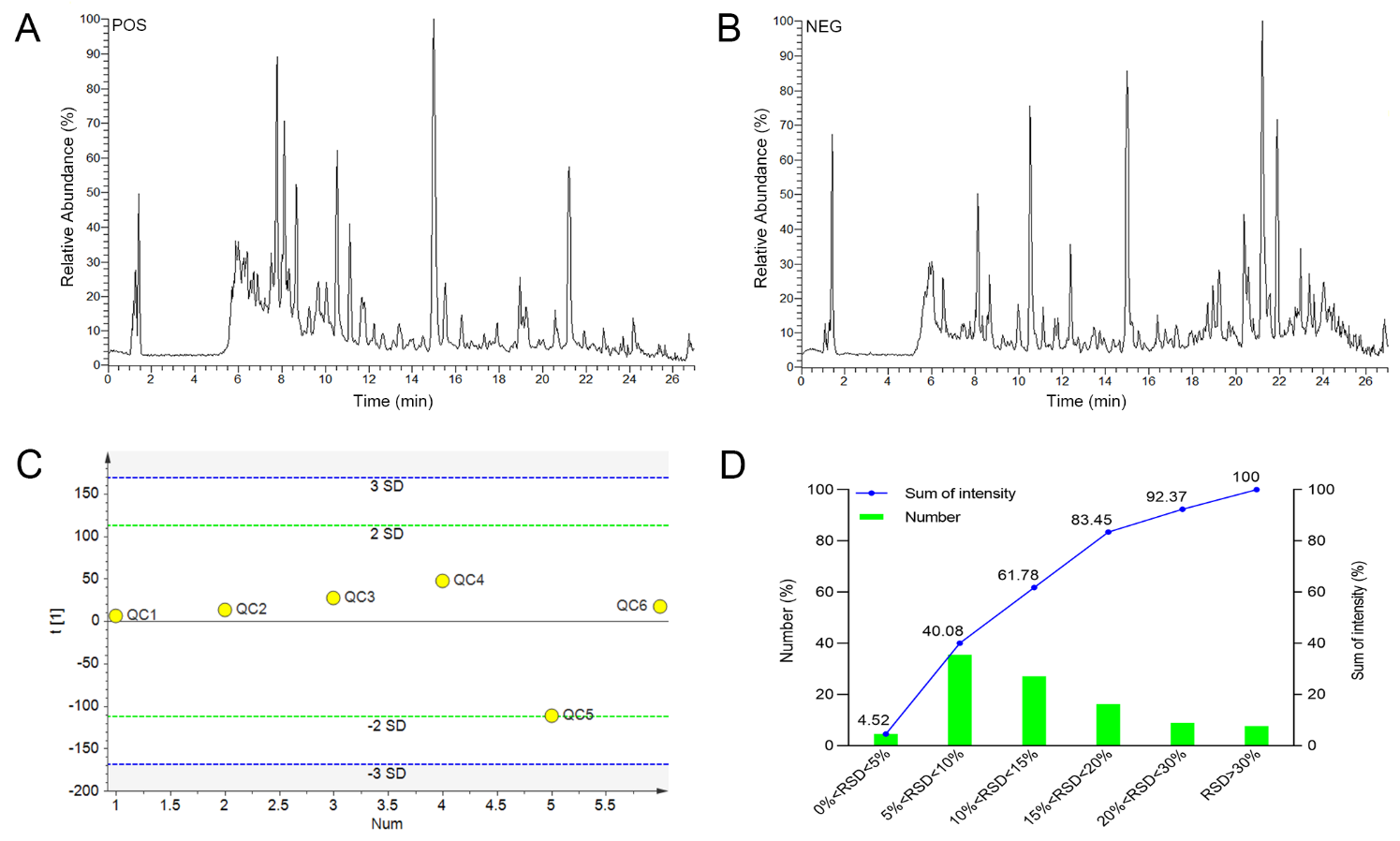


**Supplementary Figure 1.** The reliability investigation of the metabolomics method using Quality control (QC) samples. **(A, B)** The base peak intensity (BPI) chromatograms of QC samples in positive and negative mode. **(C)** PCA score plot of QC samples. **(D)** RSD% of all detected variables. Number (%), percentage of number of variables (the percentage of the number of variables that fall within the specified RSD range); Sum of intensity (%), percentage of total response of variables (the cumulative percentage of the number of variables within the specified RSD range).

## Supplementary Tables

**Supplementary Table 1.** Identification of bile acid metabolites by UHPLC-Q-Orbitrap MS based metabolomics.

| **VIP value** | **RT (min)** | **Identification** | **Formula** | **Deviation (ppm)** | **Mass fragments** |
| --- | --- | --- | --- | --- | --- |
| 20.96 | 15.02 | DCA | C_24_H_40_O_4_ | 2.85 | 785.5923 [2M+H]^+^  437.2909 [M+HCOO]^-^  391.2854 [M-H]^-^ |
| 9.06 | 10.56 | HDCA | C_24_H_40_O_4_ | 3.87 | 783.5783 [2M-H]^-^  437.2912 [M+HCOO]^-^  391.2858 [M-H]^-^ |
| 7.51 | 8.15 | ω-MCA | C_24_H_40_O_5_ | 2.67 | 373.2737 [M-2H_2_O+H]^+^  355.2631 [M-3H_2_O+H]^+^  337.2516 [M-4H_2_O+H]^+^  815.5684 [2M-H]^-^  453.2863 [M+HCOO]^-^  407.2803 [M-H]^-^ |
| 7.29 | 10.54 | UDCA | C_24_H_40_O_4_ | 2.59 | 437.2911 [M+HCOO]^-^  391.2853 [M-H]^-^ |
| 6.40 | 8.70 | β-MCA | C_24_H_40_O_5_ | 2.55 | 817.5814 [2M+H]^+^  431.2764 [M+Na]^+^  373.2735 [M-2H_2_O+H]^+^  355.2631 [M-3H_2_O+H]^+^  337.2522 [M-4H_2_O+H]^+^  453.2862 [M+HCOO]^-^  407.2802 [M-H]^-^ |
| 5.40 | 8.77 | CA isomer | C_24_H_40_O_5_ | 3.44 | 453.2860 [M+HCOO]^-^  407.2806 [M-H]^-^ |
